# Applications of artificial intelligence in nuclear medicine^☆^

**DOI:** 10.1016/j.zemedi.2026.04.004

**Published:** 2026-05-10

**Authors:** Lena Kaiser, Thomas Wendler, Deni Hardiansyah, Dirk Hellwig, Philipp Lohmann, Stephan Nekolla, Laszlo Papp, Philipp Ritt, Lalith Kumar Shiyam Sundar, Johannes Tran-Gia, Artem Zatcepin, Isabelle Miederer

**Affiliations:** a Department of Nuclear Medicine, LMU University Hospital, LMU Munich, Munich, Germany; b Diagnostic and Interventional Radiology and Neuroradiology, University Hospital Augsburg, Augsburg, Germany; c Digital Medicine, University Hospital Augsburg, Neusaess, Germany; d School of Computation, Information and Technology, Technical University of Munich, Garching bei München, Germany; e Medical Physics and Biophysics, Physics Department, Faculty of Mathematics and Natural Sciences, Universitas Indonesia, Depok, Indonesia; f Department of Nuclear Medicine, University Hospital Regensburg, Regensburg, Germany; g Partner Site Regensburg, Bavarian Cancer Research Center, Regensburg, Germany; h Medical Data Integration Center (MEDIZUKR), University Hospital Regensburg, Regensburg, Germany; i Institute of Neuroscience and Medicine (INM-4), Forschungszentrum Jülich, Jülich, Germany; j Department of Nuclear Medicine, RWTH Aachen University Hospital, Aachen, Germany; k Department of Nuclear Medicine, Klinikum rechts der Isar der Technischen Universität München, Munich, Germany; l Center for Medical Physics and Biomedical Engineering, Medical University of Vienna, Vienna, Austria; m Friedrich-Alexander-Universität Erlangen-Nürnberg, Chair for Clinical Nuclear Medicine, 91054 Erlangen, Germany; n DIGIT-X Lab, Department of Radiology, LMU University Hospital, LMU Munich, Munich, Germany; o Department of Nuclear Medicine, University Hospital Würzburg, Würzburg, Germany; p German Center for Neurodegenerative Diseases (DZNE), Munich, Germany; q Department of Nuclear Medicine, University Medical Center of the Johannes Gutenberg University Mainz, Mainz, Germany

**Keywords:** AI, Nuclear medicine, Radiation detection, Image reconstruction, Image enhancement, Medical image segmentation, Medical image registration, Radiomics, Computational nuclear oncology, Radiopharmaceutical therapy

## Abstract

Artificial intelligence (AI) has vast potential to reshape nuclear medicine. Applications can be found at every step of the processing workflow, including image acquisition, image reconstruction, enhancement, and registration, segmentation, extraction of image-derived biomarkers, and prognostication, both for clinical and preclinical research and routine use. Emerging perspectives include computational nuclear oncology, foundation models, biomorphic AI, and quantum AI – currently exploratory but rapidly evolving. However, clinical translation remains limited. This narrative review summarizes the current literature on the subject and highlights current developments and future research directions. As such, it is not intended as a systematic analysis; instead, it outlines potential future directions, supported by multiple examples.

Analysis of literature reveals impressive advances in the application of AI to nuclear medicine, such as improved time resolution, robust denoising, and automated lesion segmentation. Key challenges include the need for high-quality reference data, generalizability across scanners and populations, transparency and uncertainty quantification of model decisions, and compliance with evolving regulatory frameworks.

For the future, progress will require broad collaboration between AI developers from research and industry, clinicians, hospital information technology, and regulators, while structured initiatives such as joint symposia should actively include patients to ensure innovations address real needs. Education across disciplines will also be critical for building trust and competence towards fully embracing the potential of AI for better (nuclear) medicine.

## Introduction

**Fig. 1:**
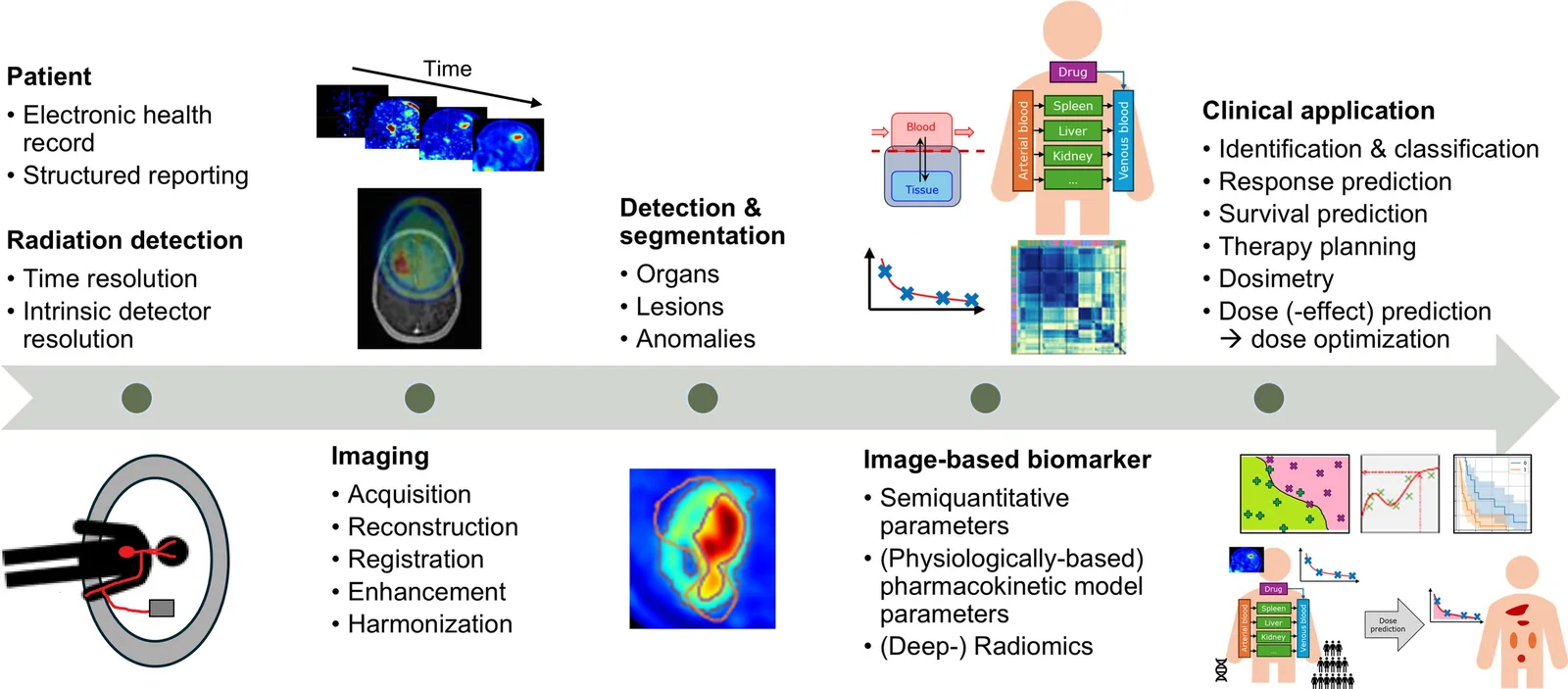
Overview of AI applications across the clinical nuclear medicine workflow.

Historically, advances in detector physics, tomographic reconstruction, tracer development, and quantitative analyses progressed largely in parallel, each contributing incremental improvements to diagnostic accuracy and therapy planning. Over the past decade, however, artificial intelligence (AI) has emerged as a unifying force, promising data-driven performance leaps in nearly every step of the nuclear medicine workflow (Fig. 1).

The transformative potential of AI is already evident in research and, in selected areas, in clinical practice. Applications such as improved photon detection, image reconstruction, enhancement, or harmonization, automated lesion segmentation, and clinical prognostication demonstrate tangible benefits in terms of image quality, workflow efficiency, and reproducibility. At the same time, AI-driven pipelines for radiopharmaceutical discovery, preclinical imaging, and digital twins for patient-tailored therapy planning highlight a rapidly expanding research frontier [1]. Yet, translation into routine clinical use remains limited, underscoring the importance of concerted development, deployment, and regulation between all involved stakeholders.

This review highlights emerging concepts and recent trends that illustrate the transformative potential of AI in nuclear medicine, without attempting to provide a comprehensive account of every publication in the field, spanning (a) radiation detection, (b) imaging (acquisition, reconstruction and processing), (c) organ, lesion and anomaly segmentation, (d) extraction of image-derived biomarkers, (e) clinical prognostic applications, (f) computational nuclear oncology, (g) preclinical research, (h) radiopharmaceutical development, and (i) regulatory aspects, as well as (j) future perspectives including foundation models, biomorphic AI, and quantum AI (Fig. 1). With such a broad approach, we extend and update recent reviews in the field of AI for nuclear medicine [2], [1], [3], [4], [5], [6], [7], [8], [9], [10], [11]. In addition to direct AI applications, we refer to computational approaches in nuclear oncology, as machine learning and deep learning are promising tools in this field. Throughout, we emphasize not only technical progress but also the cross-cutting challenges of reference data, reproducibility, explainability, as well as ethical and legal aspects, which will determine whether AI evolves from a promising research tool into a trusted clinical companion [12], [13], [14], [6].

## Prerequisites for the development and evaluation of AI tools

**Fig. 2:**
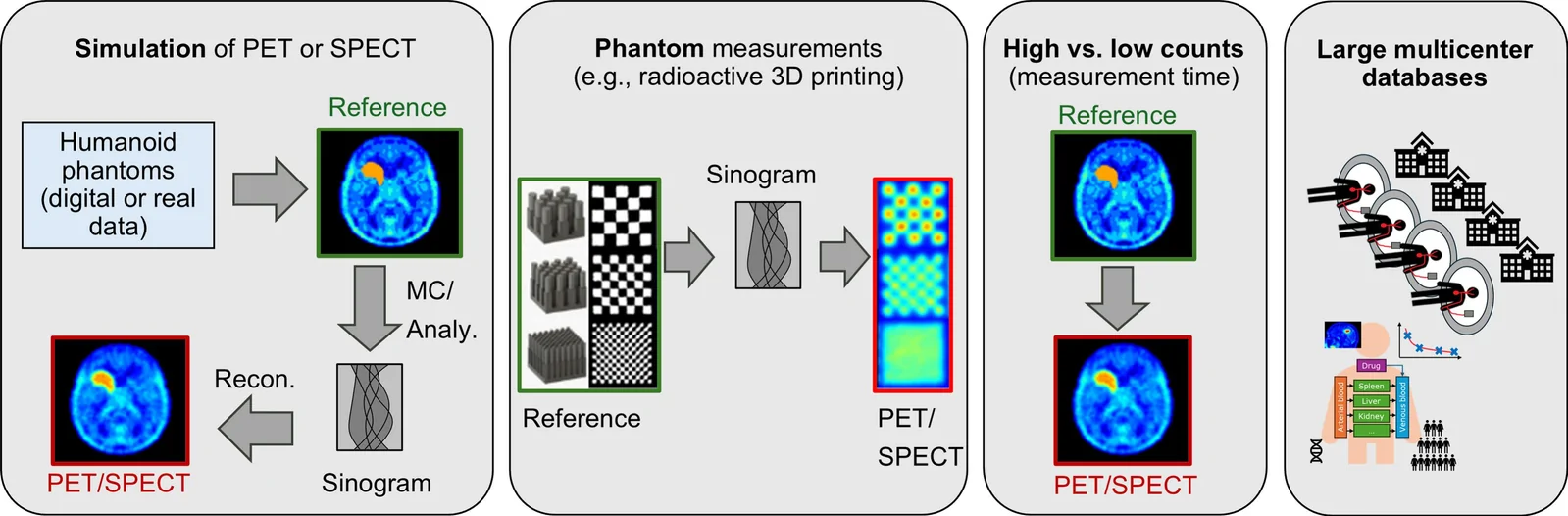
Reference data strategies for training and validation of AI models in nuclear medicine, including digital phantoms and simulations, phantom measurements, clinical acquisitions with variable protocols, and large multicenter patient databases.

A key enabler across all domains of AI in nuclear medicine is the availability of reference data, as the model performance depends on the quality of the training data. Training and validation of AI models requires high-quality, standardized large datasets that capture the variability of different patient populations, scanning devices, and data processing methods, thereby avoiding selection bias. Several strategies have been developed to meet this need (Fig. 2):

• Digital phantoms such as XCAT [15]o r real patient data, which enable the creation of realistic reference activity distributions, can be combined with Monte Carlo [16], [17], [18] or analytical simulations [19], [20] to generate synthetic imaging data under controlled conditions.
• Phantom measurements, increasingly supported by advances like radioactive 3D printing, provide experimental datasets that closely mimic real acquisition physics while remaining reproducible [21], [22], [23], [24].
• Clinical studies comparing long versus short acquisition protocols establish reference images with authentic patient anatomy but varying noise levels, supporting tasks such as denoising and harmonization.
• Large, multicenter patient databases for tasks such as outcome prediction, biomarker discovery, or building digital twins for personalized therapy. These datasets must combine imaging with clinical, biological, and demographic information to ensure robustness and fairness across populations, i.e., across underrepresented groups, such as certain age or ethnic groups. Importantly, heterogeneity in disease stage, prior treatment, tumor burden, comorbidities, and other intrinsic or extrinsic factors must be captured and considered either as a stratification variable at the study design level, via covariate-informed model integration, or as an input parameter during model application. Failure to appropriately address such heterogeneity may compromise model generalizability. Standardized data models are needed to integrate imaging with longitudinal electronic health record-derived variables such as laboratory values, prior therapies, toxicity events, pathology, outcome data, and their temporal relationships to imaging and treatment. Such harmonization should include clear definitions of minimum datasets, acquisition metadata, reconstruction settings, annotation standards, and data-sharing formats, because only then can multicenter datasets support robust training, external validation, and digital-twin applications.

Another crucial aspect is the quality of the annotations that serve for the definition of the “ground truth”. Since segmentation or classification is often performed by physicians, subjective differences can lead to inconsistencies. In addition, the heterogeneity of the data has a strong impact on generalizability: a model trained only on data from one center or from a specific type of scanner can only be applied to deviating clinical scenarios to a limited extent. For the annotation of patient data, different approaches have been proposed to mitigate biases and both intra- and inter-reader variability, as well as dealing with the fact that a real ground truth is not available for most applications. Common annotation strategies include:

• The involvement of multiple readers and blinded annotations, coupled with standardized protocols for annotation, is generally the first and likely stronger step towards reliable annotation. Double or multiple independent reads in a blinded fashion that follow the same criteria minimize bias and enable the quantification of intra- and inter-reader agreement [25]. Furthermore, Backhaus et al., showed that observer training can improve intra- and inter-observer reproducibility of extracted features, with intra-class correlation coefficients increasing from >0.86 and >0.81 to >0.91 and >0.92 after a one-hour training session [26]. Such label uncertainty is highly relevant for AI because annotation errors and inconsistent labelling propagate directly into model training and evaluation [27]. Newer AI approaches attempt to incorporate inter-reader differences directly into model development, for example, by conditioning models on annotation style to adapt to different labeling conventions [28] or by jointly learning both the expert consensus and annotator-specific labeling behaviour [29], [30].
• The use of consensus pipelines building upon multiple reads is often used to deal with outliers and better understand agreement and biases [31], [27], [32].
• The evaluation of annotations over several layers of quality control further increases control; such layers could be a peer review after initial annotation, followed by an expert validation, which aims to reduce errors further [33], [34].

Together, these approaches form the foundation for supervised training, benchmarking, and regulatory validation. Without such reference data, claims of AI performance remain difficult to verify or generalize across institutions. Consequently, reproducibility, data harmonization, and multicenter validation emerge as central themes throughout this review.

## Radiation detection

Detector technology plays a central role in PET and SPECT imaging. By leveraging the raw sensor waveforms of the detectors, AI methods have the potential to enhance timing, spatial, and energy resolution, ultimately improving image quality and diagnostic performance [35], [36]. The following examples illustrate these developments.

### Coincidence time resolution

**Fig. 3:**
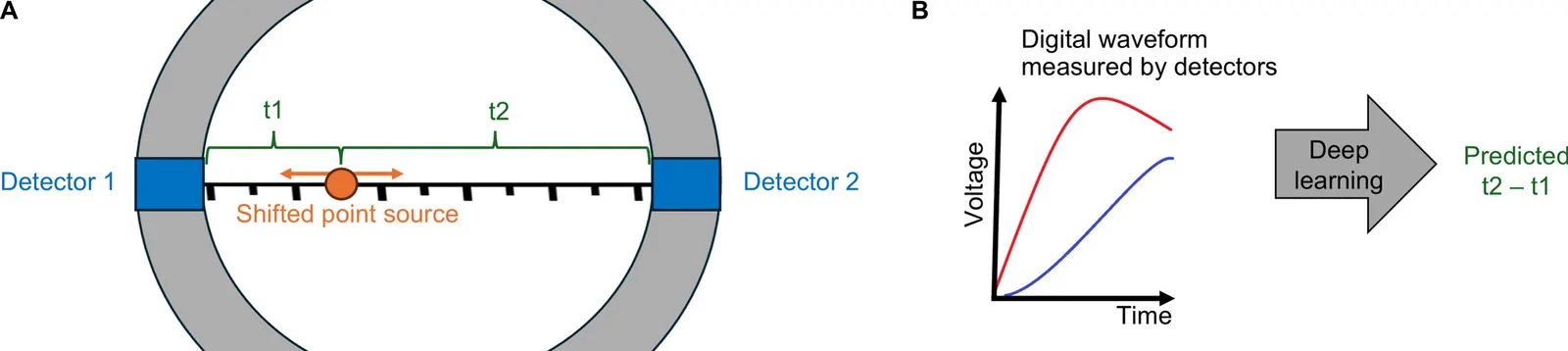
Coincidence time resolution in PET. A: generation of reference data based on known positions of a point source within the scanner. B: Digital waveforms measured by the detectors are used as input for a deep learning-based prediction of the coincidence times, enabling precise localization of the annihilation position.

Time of flight (TOF) in PET enables the use of the arrival time of annihilation photons in detectors to draw conclusions about the annihilation location. The resulting localization uncertainty Δx≈(c·Δt)/2 couples spatial resolution Δx to coincidence timing resolution Δt. By improving the timing resolution of detector technology, the spatial resolution has already been significantly improved. It is expected that the timing resolution can be further optimized by utilizing AI methods [37], [36], [38](Fig. 3).

For example, Berg et al., demonstrated that convolutional neural networks (CNNs) can accurately regress the time difference between annihilation photons, surpassing the capabilities of traditional methods. Reference data for training the model was obtained by moving a point source along a line-of-response. The learning data consisted of the known positions of the point source, decoded by the corresponding Δt, and the measured detector signals. The learned model achieved a mean Δt of 185 ps, outperforming leading-edge discriminators (231 ps) and constant-fraction discriminators (242 ps) [37]. With c ≈ 30 cm/ns, improving time resolution from 231 ps to 185 ps reduces the localization error of TOF imaging from ∼3.5 cm to ∼2.8 cm along the LOR.

### Intrinsic detector resolution

Some PET scanners traditionally employ pixelated lutetium–yttrium oxyorthosilicate (LYSO) crystals that map one-to-one onto silicon photomultipliers. However, the crystal size is limited by the size of the detector. Alternative approaches include multiplexing, where light from small crystals is distributed to several detectors via an optical fiber, and monolithic crystals, where the interaction position in the crystal must be determined based on the light distribution among silicon photomultiplier arrays. This is a more complex task that has been solved using AI methods [39], [40], [41], [42].

**Fig. 4:**
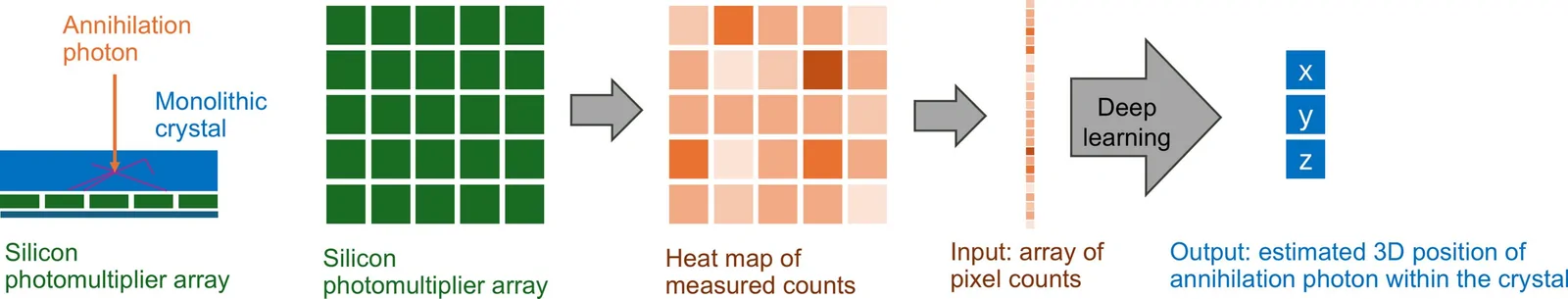
Intrinsic detector resolution with monolithic scintillation crystals. Deep learning-based prediction of the 3D photon interaction position within the crystal from the measured light distribution pattern.

For example, 42 used a neural network that ingests the number of photons detected within each silicon photomultiplier (“heat maps”) as input to predict the 3D position (x, y, z coordinates as outputs) within the crystal (Fig. 4). Reference data were generated with the GEANT4 Monte Carlo simulation toolkit. In comparison to the classic Anger logic positioning algorithm, a significant improvement in full-width-at-half-maximum resolution was achieved, from 0.96 mm to 0.78 mm at the center and from 1.6 mm to 1.15 mm at the edge of the detector, without any hardware modification.

### Outlook

In 2020, the community articulated the “10‑ps PET challenge,” noting that a time resolution below ten picoseconds would confine the TOF blur to less than a millimeter and nearly negate its impact on image quality [43]. In addition to spatial resolution, energy resolution is also critical because it governs scatter rejection. AI-driven energy estimation from waveforms is comparatively underexplored.

Further progress will require novel scintillators and detector designs combined with AI‑aided technology. Joint networks that predict time-stamp, interaction position, and photon energy in a single forward pass promise holistic optimization [36]. Additionally, AI-aided simulations could be employed to automatically optimize detector geometries and material combinations, thereby achieving the optimal balance of energy, time resolution, and cost.

A final area of application for AI in radiation measurement technology is AI-based quality control. For instance, continuous monitoring of raw data could enable the detection of anomalies or support error diagnosis. Since measurement technology itself forms the basis for precise imaging, advances in this area are essential.

## Imaging

AI also plays a pivotal role in image acquisition, reconstruction, enhancement, harmonization, and registration. AI-based reconstruction approaches range from direct generation of images from raw data to parametric reconstruction of kinetic maps. In addition, AI-driven registration strategies enable motion correction, multimodal alignment, and longitudinal analyses, which are essential for quantitative and reproducible imaging, particularly for improved diagnosis, dosimetry, and follow-up. Further methods address image enhancement, including partial volume correction and denoising, followed by harmonization techniques that ensure comparability across scanners and centers.

### Image acquisition

AI can shorten acquisition by learning to recover missing information from sparser data or shorter acquisition durations – i.e., shorter time per angle in SPECT (or faster gantry rotation), shorter time per bed position in PET, or shorter frame durations in dynamic scans. Two families of approaches are prominent: (a) projection-domain completion of sparsely acquired data and (b) image-domain restoration (section 3.4.2 for denoising).

• Projection/intermediate-view synthesis: In SPECT, AI can be used to generate synthetic intermediate projections from raw projection datasets acquired with fewer than the typical number of projections. By restoring missing projections, reconstructions with less undersampling artifacts can be achieved. Ryden et al., showed that creating 3x30 synthetic SPECT projections from 30 projections and using the total of 120 projections improved the structural similarity index metrics (against full 120 acquired projections) from 0.989 to 0.993 and increased the peak signal-to-noise ratio from 47.2 to 49.5 compared to a reconstruction using 30 projections alone [44]. A simulation study by Leube et al., (training n = 9,000, validation n = 500, test n = 500) showed good agreement between simulated projections before adding noise and synthetic projections generated from a subset of noisy input projections using AI with structural similarity index measures above 0.97[45].
• Temporal undersampling: In both SPECT (less angular projections or shorter time per projection) and PET (shorter acquisition time per bed position), accelerated acquisitions can be combined with AI-based reconstructions to maintain task performance (organ-based activity quantification, lesion detectability, kinetic parameter bias).
• Image-domain restoration. As an alternative or complement to sparse sampling, post-reconstruction AI denoising can recover image quality from short/low-count acquisitions, which is described in more detail in section 3.4.2.

When deploying acceleration, task-based evaluation is recommended (e.g., lesion detectability, error in quantitative parameters) in addition to image-quality metrics (e.g., structured similarity index measure) to confirm clinical usefulness [46].

### Image reconstruction

During conventional iterative reconstruction, a sinogram is calculated from the currently estimated image and compared with the actual measured sinogram, thereby adjusting the current estimate of the image. This has the disadvantage that no reference data from the true activity distribution and the corresponding measured sinogram are used at any point. AI methods are, by design, well-suited to bridge this gap [47], [48], [49], [50].

#### Direct reconstruction

One approach for AI-based image reconstruction is direct reconstruction using paired reference data. Here, the network learns directly how to create an optimal image from the raw data [51], [52], [53], [54].

One of the earliest frameworks, “DeepPET” employed a convolutional encoder-decoder neural network (U-Net) to reconstruct PET images directly from sinograms (training n = 245, validation n = 52, test n = 53). The group used a deformable humanoid XCAT phantom as a reference and simulated this reference activity distribution with a tool called PETSTEP [52], [53]. They realized different noise levels and thus simulated a variety of image qualities. Because the U-Net-based reconstruction was applied to 2D layers, they had a correspondingly large amount of training data. Notably, lesions and other disease-related changes were not simulated, limiting immediate transfer to oncology tasks without lesion priors. The authors demonstrated that DeepPET improves the homogeneity of activity distributions within different organs compared to conventional filtered back projection (FBP) or ordered subset expectation maximization (OSEM) reconstruction. Yet, smaller structures could only partially be restored or even disappeared completely.

Building on this concept, DirectPET introduced a large neural network architecture with a Radon inversion layer, enabling the fast reconstruction of full multi-slice PET volumes and showing feasibility for low-dose acquisitions [55]. Hashimoto et al., applied deep image prior methods for direct PET reconstruction without labeled training data, achieving better image quality than FBP or maximum likelihood expectation maximization (MLEM) in synthetic [^18^F]FDG brain studies [56]. Rashid et al., applied conditional GANs to map PET sinograms to reconstructed images, achieving lower bias and variance than FBP or MLEM while preserving quantitative accuracy [57].

Another promising direction is the incorporation of physics-informed priors or model-based deep learning to entangle the strengths of conventional system modeling with data-driven learning. For instance, unrolled networks or plug-and-play deep priors embed the forward and backprojection operators (together with Poisson noise models, normalization, and scatter/attenuation corrections) into the network architecture or loss function, thereby enforcing consistency with the physics of PET acquisition [58]. The hybrid approach mitigates the risk that purely data-driven mappings violate physical constraints or degrade quantitation when encountering out-of-distribution data. One concrete realization is the framework proposed by Mehranian and Reader [59], which utilizes forward-backward splitting and regularization in a deep unrolled architecture (i.e., alternating between a model-based update and a learned denoising step) to improve quantitative accuracy while preserving convergence properties.

Beyond sinogram-based approaches, recent work has explored list-mode deep image priors that respect the inherent temporal and event-level granularity of PET acquisition. Ote et al. [60] proposed a reconstruction technique that alternates between a regularized list-mode row-action update and a CNN-based deep image prior, achieving sharper reconstructions and a more favorable noise-contrast trade-off compared to naively applying deep image priors on binned sinograms [60]. Furthermore, Gong et al., embedded deep residual CNN representations within an iterative PET reconstruction framework based on alternating direction method of multipliers, essentially treating the neural network as a constrained image prior inside the iterative loop rather than a post-hoc denoiser. This approach improved quantitation and contrast-noise tradeoffs on both simulation and real hybrid PET data [51].

Parallel developments have also occurred in SPECT. Using simulated data from the Zubal brain phantom with a striatum-to-background ratio of 6:1, SPECTnet demonstrated that projection data combined with attenuation maps can be directly mapped to activity images with visually high resolution and low noise. Quantitatively, this resulted in mean ± SD values of 4.04 ± 0.93 in the striatum versus 3.18 ± 1.03 with OSEM, while background values were 0.98 ± 0.12 versus 0.98 ± 0.50 with OSEM [61]. More recently, DuDoSS proposed a dual-domain sinogram synthesis network that improved reconstruction quality in sparsely sampled SPECT acquisitions [62].

These studies highlight the promise of direct AI-based reconstruction for PET and SPECT, offering faster processing and the ability to handle low-dose or sparse acquisitions. Challenges remain, including reliable recovery of small structures, robustness across scanner hardware and protocols, and interpretability of the learned mappings.

#### Parametric reconstruction

**Fig. 5:**
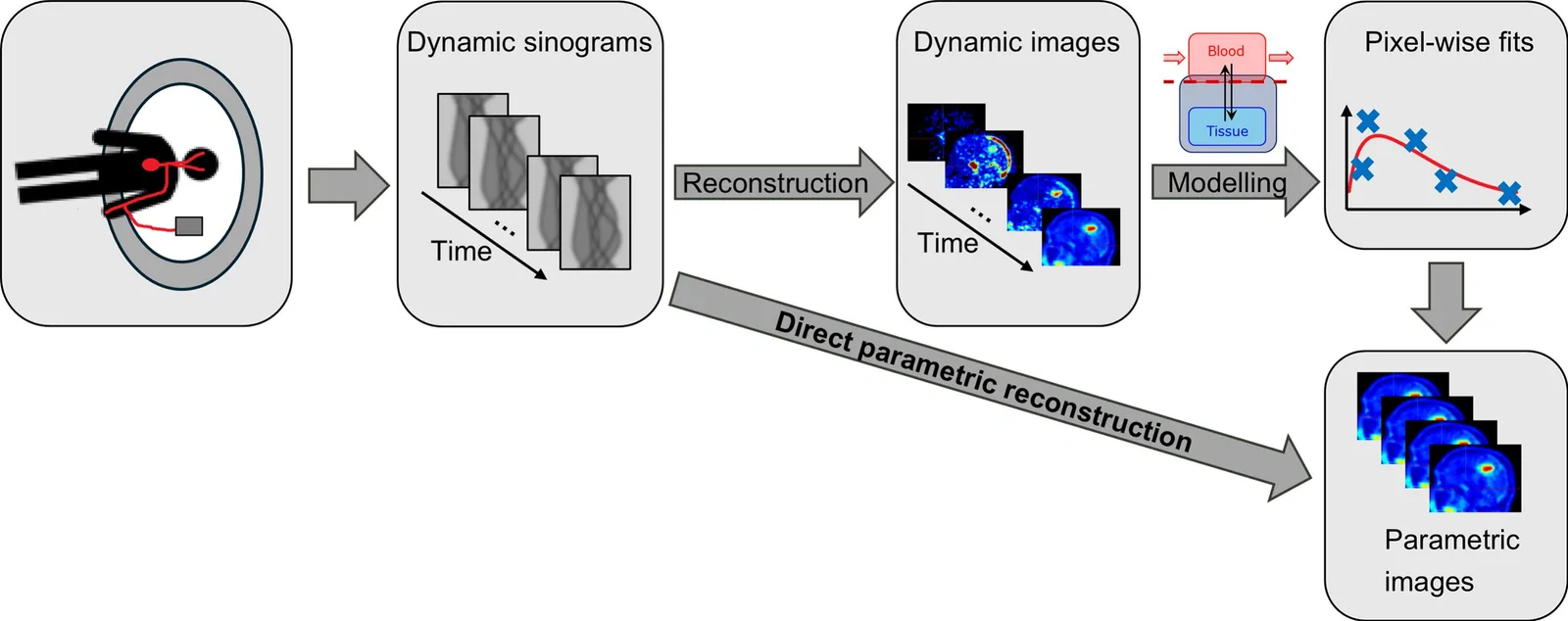
AI-based parametric PET image reconstruction. Neural networks directly generate voxel-wise kinetic parametric maps from sinograms, bypassing conventional multi-step workflows.

AI has also been used for parametric reconstruction, aiming to generate voxel-wise kinetic parameter maps directly from projection data [63], [52], [53], [64], [65], [66], [67]. In the classic workflow for kinetic modeling, dynamic data is usually reconstructed first, followed by the extraction of voxel-wise time-activity curves and final fitting of kinetic models to obtain parametric images. With AI methods, the idea is to skip the individual steps and generate the parametric images directly from the sinograms (Fig. 5).

An interesting approach for parametric reference data was proposed by the same group that established the DeepPET reconstruction [52], [53]. A dynamic 3D brain phantom was created, and the pharmacokinetic properties of the different structures were defined using a 2-tissue compartment model. Three tumors of different sizes were also inserted into this model. Subsequently, the analytic simulation tool dPETSTEP [19], [20] was used to create realistic sinogram data and corresponding PET images. This approach can also be applied in combination with XCAT phantoms, enabling the definition of different tissue classes based on a compartment model. This would allow the direct training of networks that map sinograms to parametric images.

Several groups have since demonstrated direct parametric reconstruction in clinical contexts. Li et al., utilized AI for the direct reconstruction of parametric images [68]. Conventional parametric images were used as a reference, from which the blood input function for the kinetic model was determined via the aorta (training n = 16, test n = 4). For direct parametric reconstruction, the last five time points of the dynamic study were used as input. The network was optimized by comparing it with the image from the conventional method. Another group also used an image-generated blood input function and a parametric image from conventional methods as a reference (training n = 126 corresponding to 8365 slices, validation n = 37, test n = 40) [66]. In this case, only one static PET image was used as input. The proposed modified cycle generative adversarial network (CycleGAN) showed the lowest deviation from the ground truth data compared to other deep learning approaches. Huang et al., highlighted the benefits of long axial field-of-view PET scanners (training n = 160, validation n = 20, test n = 20; 10-fold cross-validation), where increased sensitivity enhances dynamic signal-to-noise ratio and supports AI-regularized direct parametric imaging [65].

### Image registration

Image registration is required on the one hand for the superimposition of multimodal data, for longitudinal image analysis, and for motion correction, and therefore naturally follows reconstruction. The former two play a central role in multimodal image analysis, dosimetry, and follow-up of disease development. The latter is particularly important for respiratory, cardiac, and any other type of patient movement during the scan, resulting in better quality images.

There are various hardware and data-driven approaches to enable motion correction [69]. Long short-term memory (LSTM) networks have been applied to correct for breathing [70]and cardiac [71] motion. Deep learning-based strategies have also been explored for head motion correction, where Tumpa et al., demonstrated that their approach could match the accuracy of established non-AI algorithms while substantially reducing runtime [72]. Data-driven image registration has also been performed as part of direct image reconstruction [52], [53]or combined with automatic machine learning-based segmentation [73].

Kim et al., proposed an unsupervised deep learning framework (22 datasets used for training/internal validation, 14 datasets used for external validation) for deformable multimodal registration of SPECT and CT images, outperforming traditional optimization-based methods in speed (6 s vs. 50 s) and robustness (voxel-wise RMSE reduced by 27% for kidney and 33% for tumor) [74]. Similarly, Schaefferkoetter et al., successfully applied deep learning-based elastic registration between PET and CT images to improve PET/CT attenuation correction [75].

Huff et al., developed an automated lesion-matching method for longitudinal PET/CT scans in cancer patients, achieving performance comparable to that of two expert readers [76]. The system substantially reduced the time burden in high-disease-burden cases (median matching time reduced to 3.9 min from 60 and 30 min by two nuclear medicine physicians) and enabled efficient, reproducible lesion tracking across multiple time points, supporting clinical adoption for more comprehensive monitoring.

While many AI-based image registration promise high efficiency and scalability, the main challenge highlighted by 77is the availability of ground-truth spatial transformations. The advantage of nuclear medicine in comparison to morphological imaging might be that digital phantoms and simulations can be applied to generate such reference data.

### Image enhancement

After reconstruction and, when needed, registration, AI can further improve image quality through enhancement strategies such as partial volume correction, noise reduction, and harmonization. These methods aim to counteract physical limitations of the imaging system and acquisition protocols, thereby increasing diagnostic reliability and quantitative accuracy [78].

#### Partial volume effect correction

The limited spatial resolution of PET and SPECT leads to partial volume effects (PVE), where activity from small structures is blurred into neighboring regions. Deep learning-based partial volume correction (PVC) has been proposed to address this issue. Leube et al. trained an U-Net to learn the SPECT-related resolution loss introduced by collimation in combination with a noncircular detector orbit, which together produce a highly anisotropic, distance-dependent point spread function (PSF) [46]. The network was optimized on paired data so that, given a reconstructed SPECT image affected by this anisotropic PSF, it predicts a PVC-corrected estimate of the underlying activity distribution. With realistic Monte Carlo-based training data artificially created using the XCAT phantom (training n = 10,000 and test 500 random activity distributions), performance was compared against iterative Yang PVC [79], [80], with the deep learning approach more closely matching the reference activity distribution and outperforming the iterative method (fraction of voxels with relative activity concentration deviation <5%: improved to 35.8% vs. 15.1%).

#### Denoising

Beyond PVC, noise reduction techniques are needed to increase diagnostic and prognostic performance. Numerous methods for noise suppression exist outside of nuclear medicine [81]. Noise suppression is particularly relevant in low-count situations such as dynamic studies with short frame durations, in pediatric applications to reduce radiation exposure, and for an accelerated image acquisition by subsampling.

Supervised comparisons of different network architectures have highlighted task-dependent trade-offs. For instance, Wang et al., compared five different deep learning networks for image denoising [82]. Reference data were generated using both short and long measurements, corresponding to different counting statistics and noise levels (32 scans from 21 patients, external test: 20 scans from 10 patients). The authors point out that a single solution for all applications of interest may be difficult to achieve, even within the same patient using the same tracer. They presented an example image in which a small liver lesion was visible after four epochs; however, the brain structures remained blurred. While the brain structures were very sharp after 24 epochs, the small lesion in the liver disappeared due to excessive smoothing. Therefore, task-based evaluation (lesion detectability, bias of quantitative parameters) should complement peak signal-to-noise ratio and structured similarity index matrix, particularly when denoising is used as an acceleration strategy (short scans/low dose).

Another approach is the application of denoising diffusion probabilistic models [83]. In these, Gaussian noise is gradually added (forward transformation), and then high-resolution PET images are iteratively regenerated (training n = 302, validation n = 15, various test set configurations n = 10-60). This approach promises very reliable noise reduction. Yu et al., presented that this method allows for the restoration of a homogeneous activity distribution within organs, while small lesions remain sharp and visible. A disadvantage of denoising diffusion probabilistic models is that they require a significant amount of computing power and do not allow for rapid denoising.

The advantages and disadvantages of the different generative methods were highlighted by 84. GANs allow fast sampling and produce sharp images but may struggle – depending on configuration – with non-average, disease-related changes. Variational autoencoders maintain data diversity and are fast, yet reconstructions often appear blurred. Denoising diffusion models can yield sharp images while preserving diversity; the trade-off is typically slower sampling. The authors propose a hybrid acceleration approach, utilizing GANs to expedite the diffusion denoising steps. In nuclear medicine, these generative methods have been applied to PET and SPECT denoising for acquisition-time reduction, with evaluations emphasizing lesion detectability and kinetic accuracy in addition to standard metrics [39], [40], [50], [85], [86].

### Image harmonization

Harmonization is essential for multicenter evaluations and studies because it enables pooling of data, external validation, and fair model comparison across scanners, protocols, and sites – while preserving disease signal and quantitative readouts. Approaches fall broadly into two categories: (a) physics or protocol harmonization (e.g., calibration, attenuation/scatter correction settings, reconstruction presets) applied at acquisition and/or reconstruction time, and (b) data-driven image-level harmonization or domain adaptation that mitigates residual inter-site differences [87].

At the physics layer, scanner calibration, attenuation, and scatter correction settings, and standardized reconstruction protocols are essential. In PET/CT, EARL accreditation (EANM Research Ltd.) provides standardized calibration and reconstruction protocols to harmonize SUVs across sites [88]. Any AI image-level harmonization should build on, not replace, EARL-compliant pipelines. For absorbed dose-related tasks and quantitative SPECT/CT (e.g., for Lutetium-177), physics-layer harmonization and camera calibration remain indispensable before any AI-based post-processing [89].

Data-driven harmonization often leverages generative models (e.g., style transfer) to match site-specific noise or textural characteristics while attempting to preserve quantitation [87]. As an unpaired example, Haberl et al., [90]trained a CycleGAN to translate images between center “styles” using cycle consistency and reported improved identification of distant metastasis. They also observed boundary artifacts and occasional hallucinations – known risks associated with GANs – underscoring the need for careful quality control. Paired or semi-paired strategies, when feasible, can reduce hallucination risk but are harder to assemble at scale.

Robust harmonization workflows require numeric and visual safeguards. For example, pre- and post-numeric checks (e.g., liver SUV/SUL for PET or Bq/ml stability for SPECT) and blinded visual review (for diagnostic imaging) should be applied to ensure anatomy and lesion conspicuity are preserved. Ultimately, harmonization must be judged on downstream endpoints such as lesion detectability, reproducibility of radiomic features, and the absence of kinetic or dosimetric bias – in line with evaluation practices in RELAINCE [91], the EANM/SNMMI radiomics guideline [92], CLEAR [93], and CLAIM [94].

## Detection and segmentation of organs and lesions

The next step in this workflow is image segmentation, where regions of interest are identified and delineated as a prerequisite for quantitative assessment. From the physician’s perspective, the value of automated segmentation lies in enabling automatic quantification within optimized clinical reporting pipelines. AI-derived measurements, such as whole-body tumor burden, lesion counts, lesion shape and size, organ-based uptake summaries, or longitudinal changes, could enrich structured reports and improve the quality of progression metrics, such as PERCIST [95]. AI-based methods, such as nnU-Net (no new U-net) or nnDetection, have become the established standard for detecting and segmenting lesions [96], [97]. They enable the tumor volume or tumor burden to be determined objectively and reproducibly. Both approaches are fully automated workflows that adapt data pre-processing, augmentation, architecture adaptation, and training setup itself. Of particular relevance is the use of nnU-Net for full body organ segmentation, as implemented, for example, in TotalSegmentator for CT and MRI [98]or MOOSE [99] for PET/CT.

In oncology, the definition of tumor volume and tumor burden is important for therapy planning and the assessment of therapy response or progression [100]. A practical example is the automatic segmentation of brain tumors in amino acid PET images. For training of an nnU-Net, Gutsche et al., used a clinically established intensity threshold as segmentation reference, which was calculated based on the signal in healthy brain tissue on the contralesional side [101], yielding good results. Yet, a follow-up study on an external test cohort presented a systematic underestimation of automatically segmented volumes when using the trained models from the first center [102]. This confirms that scanner and reconstruction variability impact segmentation performance, as well as the threshold-based definition of reference segments. A very similar approach was presented by another group, which applied nnU-Net to patients with neuroendocrine neoplasia [103]. Also in this study, the reference segmentation was defined using a thresholding approach based on the healthy liver signal.

Segmentation-derived volumetrics – metabolic tumor volume and total lesion glycolysis – have been widely shown to re-stratify risk and prompt treatment adaptation in prospective and large retrospective studies. In metastatic non-small cell lung cancer and melanoma receiving systemic therapy, higher metabolic tumor volume predicts inferior outcomes and is used to escalate or combine therapies in many centers [104], [105]. In lymphoma, baseline total metabolic tumor volume robustly identifies high-risk biology and is being operationalized for trial-level intensification/de-escalation; recognition of method variance has prompted an international benchmark to standardize segmentation and cut-offs [106].

### PET segmentation challenges

Despite recent advances, AI-based segmentation in PET lags behind CT and MRI, primarily due to the lack of large, publicly available, and comprehensively annotated datasets. While radiology benefits from more than a million open studies for model development and foundation models, PET lesion segmentation datasets cover only about 2,000 cases, primarily derived from three challenges: AutoPET, HECKTOR, and DeepPSMA.

AutoPET provided ∼1,600 PET/CT scans annotated for whole-body lesions across lung cancer, lymphoma, melanoma, and prostate cancer [107]. To evaluate the generalizability of the models for multi-center and multi-tracer data, two different tracers were used at two different centers. One of the best results was achieved by a model that utilized anatomical CT information in conjunction with the PET signal [108]. The PET information was first used to classify which tracer was involved. This was followed by the application of an nnU-Net with multimodal data input. However, the resulting Dice coefficients remained low and highly different among applications and centers.

The HECKTOR challenge, included ∼1,200 PET/CT scans from 11 institutions, enhancing geographic and scanner diversity (including Philips Gemini, GE Discovery, and Siemens Biograph scanners), but annotations were limited to head-and-neck tumors, restricting the applicability for whole body segmentation purposes [109], [110], [111].

The recent DeepPSMA challenge comprises paired [^18^F]FDG and [^68^Ga]Ga-PSMA or [^18^F]F-DCFPyL PET/CT data from 100 prostate cancer patients, annotated with standardized thresholds [112]. While unique for paired tracers and clinical context, it is a single-center challenge and focused solely on prostate cancer, with a small sample size.

These datasets are valuable but leave critical gaps: limited overall scale, narrow tracer diversity (dominated by [^18^F]FDG and PSMA ligands), underrepresentation of scanner vendors beyond Siemens and GE, and insufficient clinical metadata.

### Outlook

These developments demonstrate that AI-based segmentation is moving toward more robust, task-specific, and clinically validated workflows. Nevertheless, widespread adoption will require larger, more diverse, and better-annotated datasets, harmonized evaluation benchmarks, and rigorous external validation to ensure reproducibility across tracers, scanners, and clinical sites.

The results from PET segmentation challenges indicate that establishing a single model trained for different radiopharmaceuticals with potentially highly variable biodistribution is currently not yet solved and might require new approaches, including multi-agent systems in combination with foundation models. The recent trend is toward so-called segment anything models (SAM), which aim to achieve efficient segmentation across different modalities and disease applications by utilizing foundation models [113], [114], [115], [116], [117]. These methods should reduce the need for expert annotations and enable flexible adaptation for new tasks. Yet, the performance of such models remains limited [117], [118].

Another very recent approach utilizes an extended version of the traditional long short-term memory model (xLSTM) as a backbone within a U-Net for medical image segmentation, enabling improved handling of long-range correlations while capturing local properties in both the spatial and temporal domains [119], [120], [121], [122]. Initial results on CT, MRI, or dermoscopic images showed a superiority for complex segmentation tasks over other methods. A potential benefit for nuclear medicine tasks is yet to be evaluated.

## Pharmacokinetic modeling

**Fig. 6:**
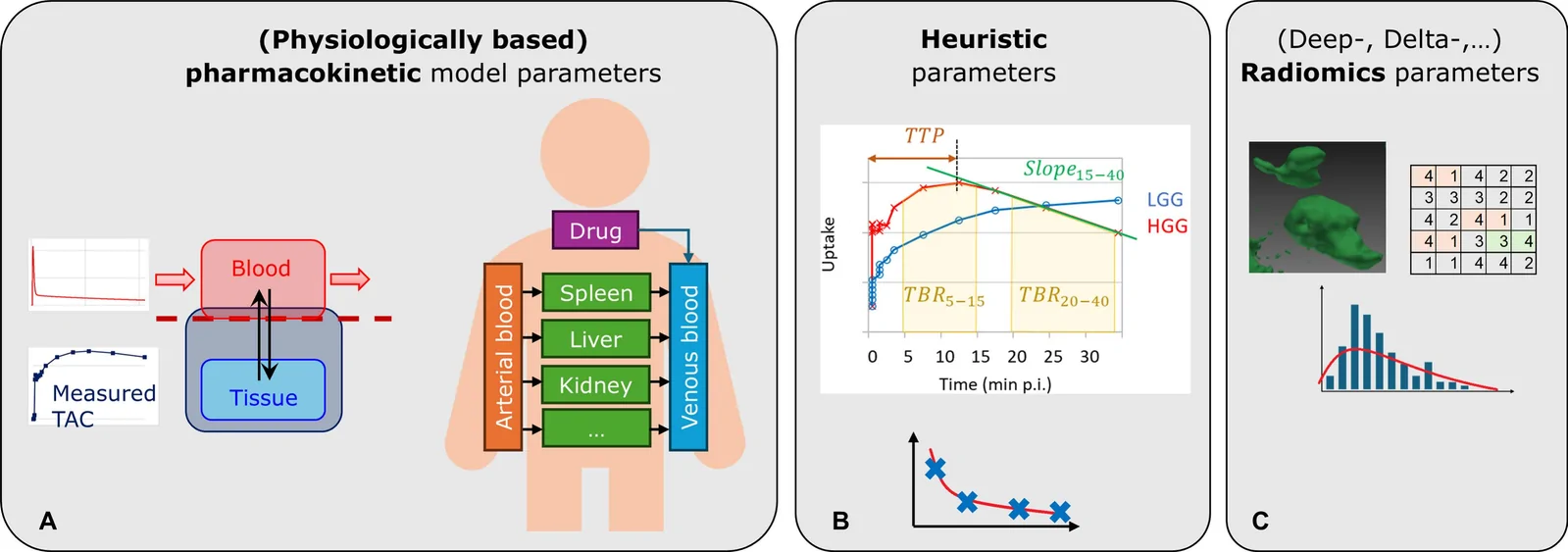
Examples of image-derived biomarkers: A: quantification of pharmacokinetic properties. B: quantification using heuristic descriptions of tracer kinetics and uptake. C: radiomic features including shape, first order, and texture parameters.

Image-based biomarkers are quantifiable features extracted from medical images that provide objective information about biological processes, disease characteristics, or responses to therapy. Multiple biomarkers can be extracted from static and dynamic molecular images and used as input for machine learning-based prediction models and clinical decision support tasks. How the markers are calculated can be split in (a) parameters derived from dynamic images for (physiologically based) pharmacokinetic models, (b) heuristic parameters providing a simplified description of kinetic and static signal properties typically inspired by expert criteria for image evaluation, and (c) automatic shape, intensity and texture-based features which run under the umbrella denomination “radiomics” (Fig. 6). In this section, we will concentrate on the potential of AI to facilitate (physiologically based) pharmacokinetic modeling.

Pharmacokinetic modeling of dynamic image sequences enables quantification of tracer kinetics and tissue properties. Traditionally, kinetic parameters are derived from compartmental models or heuristic descriptions of activity over time. These models have demonstrated clinical value in several areas, including whole-body PET staging [123], differentiation of brain tumor subtypes [124], [125], [126], and classification of subjects in neuroreceptor imaging studies [127]. Recently, artificial intelligence (AI) has been integrated into multiple stages of this process, improving parameter estimation, facilitating physiologically based pharmacokinetic (PBPK) modeling, and supporting personalized dosimetry [128], [4], [5], [129], [130].

### Image-derived input function

Obtaining an accurate arterial input function (AIF) is essential for tracer kinetic modeling but traditionally requires arterial blood sampling, which is invasive, time-consuming, and introduces workflow challenges. To overcome this, various methods have been proposed: (a) AI-based segmentation of vascular structures to extract input functions automatically, (b) curve-based prediction to predict AIFs directly from dynamic PET time-activity curves, (c) joint estimation approaches (SIME) that simultaneously estimate model parameters and parent in plasma input function. Despite these advances, current challenges and potential applications of AI include partial volume effects, motion artifacts, and low temporal sampling [131].

For brain imaging applications, De Francisci et al., introduced a method to automatically segment the internal carotid artery from MRI and use this as a start to identify the relevant voxels (region of interest, ROI) in the corresponding dynamic PET image, while compensating for motion and partial volume effect [132]. Similarly, the input function can be directly derived from the dynamic PET by segmenting the internal carotid artery (ROI) as proposed by Zanotti-Fregonara et al., in an earlier machine learning-based work [133]. Chavan et al., implemented a semiautomatic pipeline that selects the best PET frame for manual thresholding of the carotid artery, which is used as input for a modified 3D U-Net to obtain a complete carotid segmentation [134]. Both studies applied their pipelines to dynamic [^18^F]FDG PET brain scans. Chavan et al., also added a recurrent neural network-based downstream processing to account for partial volume effects. Similarly, for thorax, abdomen, or full body applications, the left heart ventricle or the aorta in the CT has been automatically segmented using supervised ML approaches and used as input function for parametric image generation as implemented by several groups for [^18^F]FDG [135], [136], [137]. If a more complex input model is required, other vessels, such as the pulmonary artery and portal vein, can be used [138].

Averaging over the resulting region of interest provides an approximation of the arterial input function. This approach, despite its simplicity, presents several challenges, including partial volume effects, motion artifacts, and the lack of a metabolite and plasma fraction correction [131]. In a [^15^O]H_2_O brain positron emission tomography (PET) study, 139used three different image-derived time-activity curves, extracted from the carotid arteries, as input for a Gaussian process-based model to predict the arterial input function. In another study, the arterial input function was directly derived from dynamic PET images using a deep convolutional neural network, using the two tracers [^18^F]FDG and [^11^C]DPA-713[140]. Recently, Moradi et al., introduced another automatic approach using a wavelet transform combined with an unsupervised clustering strategy to obtain both arterial and venous input functions of the brain for [^18^F]FDG [141].

The early work by Mikhno et al., already demonstrated the potential of simultaneous estimation of model parameters and the parent in plasma input function from multiple tissue time-activity curves in combination with machine learning-based predicted model constraints. This approach enables the completely blood-free estimation of the metabolite-corrected plasma input function [142].

### Pharmacokinetic parameter estimation

Beyond extracting input functions, machine learning has also been applied to directly estimate kinetic parameters, bypassing conventional curve-fitting approaches. These methods aim to accelerate parametric image generation, leverage spatial dependencies across voxels for regularization, and mitigate partial volume effects.

To the best of our knowledge, the first works in this direction are as recent as 2022. In a simulation study for dynamic [^18^F]FDG PET, Moradi et al., used an autoencoder setup to simultaneously denoise time-activity curves and input functions and obtain microparameters of a 2-compartment model from dynamic [^18^F]FDG PET sequences using a Gaussian process regression [143]. The same year, Huang et al., used a 3D U-Net to estimate macroparameters of the Patlak model in a supervised fashion [65]. The network took a static PET frame and used a conventionally computed irreversible uptake constant Ki image as reference. Similarly, also in 2022, Cui et al., proposed using conditional deep image prior taking as input the dynamic PET frames and a corresponding anatomical image (CT or MRI) for obtaining Logan macroparameter images in an unsupervised fashion [144].

De Benetti et al., proposed the use of a self-supervised spatio-temporal 3D U-net to obtain the parameters of an irreversible 2-tissue compartment model for [^18^F]FDG [137]. The method yielded results comparable to curve fitting, producing smoother parameter maps within solid organs at a fraction of the time. Langer et al., extended this approach to handle multiple input functions derived from the segmentation of the aorta, the pulmonary arteries, the portal vein, and the ureters from CT [138].

Recently, Tehlan et al., introduced the concept of physiologically implicit neural representations – patient-specific neural networks trained in an unsupervised fashion to calculate parametric images from dynamic [^18^F]FDG sequences [145]. While requiring more computation power than the approach of De Benetti et al., , they achieved significantly better fits (mean squared error of 0.01 ± 0.09 vs. 0.07 ± 0.3) without the need for large cohorts for training (n = 24), while keeping the remaining advantages.

Vashistha et al., used dynamic TOF-PET to directly create parametric images without the need for invasive arterial sampling, anatomical data (here brain MRI), and paired training data [146]. The LSTM-based parameter estimation showed good agreement with conventional least squares fitting (Pearson correlation coefficient >0.9) while reducing calculation time (37% faster) and significantly improving contrast-to-noise ratio (p < 0.05, paired t-test).

Ding et al., proposed the application of machine learning for the separation of region-based time-activity curves from dual tracer acquisitions using [^18^F]FDG and [^68^Ga]Ga-DOTATATE for patients with neuroendocrine tumors [147]. A multi-tracer compartment model was applied as reference for training a recurrent extreme gradient boosting model.

### Physiologically based pharmacokinetic modeling

Physiologically based pharmacokinetic (PBPK) modeling has become an essential tool in radiopharmaceutical (RPT) drug development and therapy. Unlike empirical curve-fitting approaches, PBPK models explicitly incorporate anatomical, physiological, and drug-dependent parameters, such as organ volumes, blood flow rates, transport processes, receptor binding, and elimination pathways [148]. This structure allows robust extrapolation across patient populations, radionuclides, or dosing regimens, and supports in silico testing of therapy protocols prior to clinical implementation. First in-human applications using dynamic PET have already been reported [149], [150], [151].

### Outlook

Machine learning enhances pharmacokinetic modeling at multiple stages: (a) input function estimation by replacing invasive arterial sampling with non-invasive, image-derived alternatives; (b) parameter estimation aiming to produce smoother, faster parametric images than traditional curve-fitting; (c) PBPK integration, combining physiological modeling with AI to improve predictive accuracy and explainability. However, most methods remain experimental, and widespread clinical adoption will depend on validation across diverse datasets and imaging protocols.

## Diagnostic and prognostic applications

By extracting complex imaging features, integrating them with clinical and biological data, and applying advanced predictive models, AI enables stratification of patients, forecasting of treatment outcomes, and development of decision support tools. This chapter reviews the role of radiomics, machine learning, and models for prediction, classification, and outcome assessment across oncology, neurology, and cardiology.

Beyond the quantification of pharmacokinetic properties, information from intensity histograms and texture properties derived from intensity distributions within a volume of interest, or even on a voxel basis, has been utilized in nuclear medicine for the development of prognostic and predictive models. Radiomics has demonstrated its value in nuclear medicine for various clinical applications, such as the prediction of response to therapy or the prediction of biomarker expression for targeted therapies and newer treatment options in various types of cancer, the diagnosis or prognosis in patients with neurodegenerative diseases, as well as in cardiovascular and thyroid diseases [152]. Although most radiomic studies use mathematically predefined features, sometimes referred to as feature-based radiomics, new advances such as deep learning-based radiomics [153], delta radiomics [154], or voxel-wise representation of radiomic features in feature maps [155]offer great potential for future studies and need to be investigated further, particularly in the field of nuclear medicine. Unfortunately, the clinical translation of these promising results appears to be challenging, partly due to the high variability and limited reproducibility of the findings. More detailed information on this topic is beyond the scope of this article and can be found elsewhere [156], [157], [158], [159].

### Applications in oncology

Radiomics has demonstrated its value for diagnosis and classification of cytologically indeterminate thyroid nodules based on [^18^F]FDG PET radiomics. For example, Ceriani et al., [160]developed a radiomics classifier for the prediction of the final diagnosis of [^18^F]FDG-avid thyroid incidentalomas in comparison with standardized uptake values and showed that a radiomics shape feature sphericity yielded the highest classification accuracy. A complete overview of studies investigating the value of radiomics for diagnosis and classification of thyroid diseases and other clinical conditions in nuclear medicine is beyond the scope of this article and can be found elsewhere [161], [152], [162].

Another promising area for radiomics is the prediction of response to therapy. Eertink and colleagues assessed the value of [^18^F]FDG PET radiomics features to predict treatment outcome in patients with diffuse large B-cell lymphoma [163]. Based on a large group of more than 300 patients, the combination of radiomics and clinical features showed the highest diagnostic accuracy with an area under the receiver operating characteristics curve (AUC) of 0.79. Similarly, Esposito et al., showed that a support vector machine classifier based on [^18^F]FDG PET outperformed a CT-based classification with an AUC of 0.80 compared to 0.75 for prediction of therapy response in B-cell lymphoma in a bi-centric study [164].

Differences in image-based tumor heterogeneity have been demonstrated to be of value for the prediction of survival based on somatostatin receptor PET/CT in patients undergoing radionuclide therapy by Werner and colleagues [165]. Based on the analysis of more than 800 metastases, the textural feature entropy predicted both progression-free and overall survival and demonstrated that the assessment of imaging-based heterogeneity provides prognostic information and outperformed conventional PET parameters, which failed to predict response. In contrast, early and delta radiomics features derived from [^18^F]FDG PET scans from patients with locally advanced cervical cancer could not predict survival [166].

### Applications in neurology

AI and radiomics have become an essential part of neurology, a medical discipline that heavily involves medical imaging, including nuclear imaging procedures. AI in radiomics has been investigated in particular to automate time-consuming and rater-dependent tasks such as lesion segmentation in patients with stroke, brain tumors, or other brain lesions, to predict molecular and genetic signatures based on imaging features (“virtual biopsy”) or for differential diagnosis, such as the differentiation between treatment-related changes from recurrence in patients with brain tumors. Further, AI and radiomics have been investigated for prognostication and outcome prediction in various neurological and neurodegenerative diseases, including mild cognitive impairment, Alzheimer’s disease, multiple sclerosis, or Parkinson’s disease. These technologies predict the functional outcome in stroke or the prognosis, treatment response, and risk of recurrence in patients with brain tumors. The use of AI technologies is and will be changing neurology, but large multicentric prospective studies need to be performed for a successful clinical translation of these promising technologies to aid as decision support for future neurologists. Please refer to the following articles for further details and future directions of AI in neurology [167], [168], [169].

### Applications in cardiology

The use of complex analysis approaches in cardiology in general and nuclear cardiology in particular has a rather long history due to its widespread use of myocardial perfusion imaging (MPI) in the USA since decades [170], [171]. One of the earliest references focused on the automated interpretation of planar perfusion data using neural networks in 1994 [172]. The same author, Gerald Porenta published an editorial on the use of AI in nuclear cardiology reflecting on more than two decades of academic work [173]. Although the results of last year’s research may already be outdated, this summary is still valid: validation of methods is one of the key points.

In general, the use of AI in nuclear cardiology is focused on three fields, mainly in myocardial perfusion imaging with SPECT: improving the workflow, including acquisition and motion correction, extracting quantitative information, and optimizing disease detection and predictive value of these procedures [174], [175], [176]. Of special note is that several academic centers have access to very large patient cohorts from single and multi-center studies and produce commercially available software, which makes the logistical “boundary conditions” excellent. Data analysis is further accommodated as the heart’s left ventricle can be considered a well-defined geometrical object, which can be well segmented in a highly automated way, resulting in a relatively small amount of data to be further processed. Still, as discussed in chapter 10 in more detail, the discrepancy between academic publications and actually regulatory approved products is quite striking.

### Outlook

AI has the potential to transform traditional decision tree-based diagnosis and prognosis in nuclear medicine. An essential step towards clinical applicability and regulatory approval is a proven generalizability of models across different patient populations, centers, and imaging devices. Structured and automated reporting may further facilitate a faster and broader implementation of such methods. To enhance the understanding of individual patient characteristics and diseases, and to enable more patient-specific decisions and therapies, it will be essential to incorporate heterogeneous data sources, such as imaging biomarkers, clinical variables, histopathologic and molecular genetic information, and patient history.

In this context, new approaches such as TabPFN, a model trained on huge synthetic tabular datasets, represent a key step in this direction [177], [178]. The pre-trained model can be re-trained and applied to an arbitrary unseen real-world dataset without any further adjustment. By varying the proportion of non-informative parameters or the proportion of outliers, it was shown that other methods have a significantly reduced performance, e.g., for a larger number of outliers, while TabPFN remained relatively robust. An initial application on [^18^F]FDG-PET data for predicting progression-free and overall survival in lymphoma patients highlights its potential to integrate clinical, imaging, and biological features into unified prognostic scores [113], [114], [115], [116]. Further exploration of such foundation-like models for tabular and multimodal inputs will be key to advancing decision-support tools in nuclear medicine.

## Computational nuclear oncology for radiopharmaceutical therapy

**Fig. 7:**
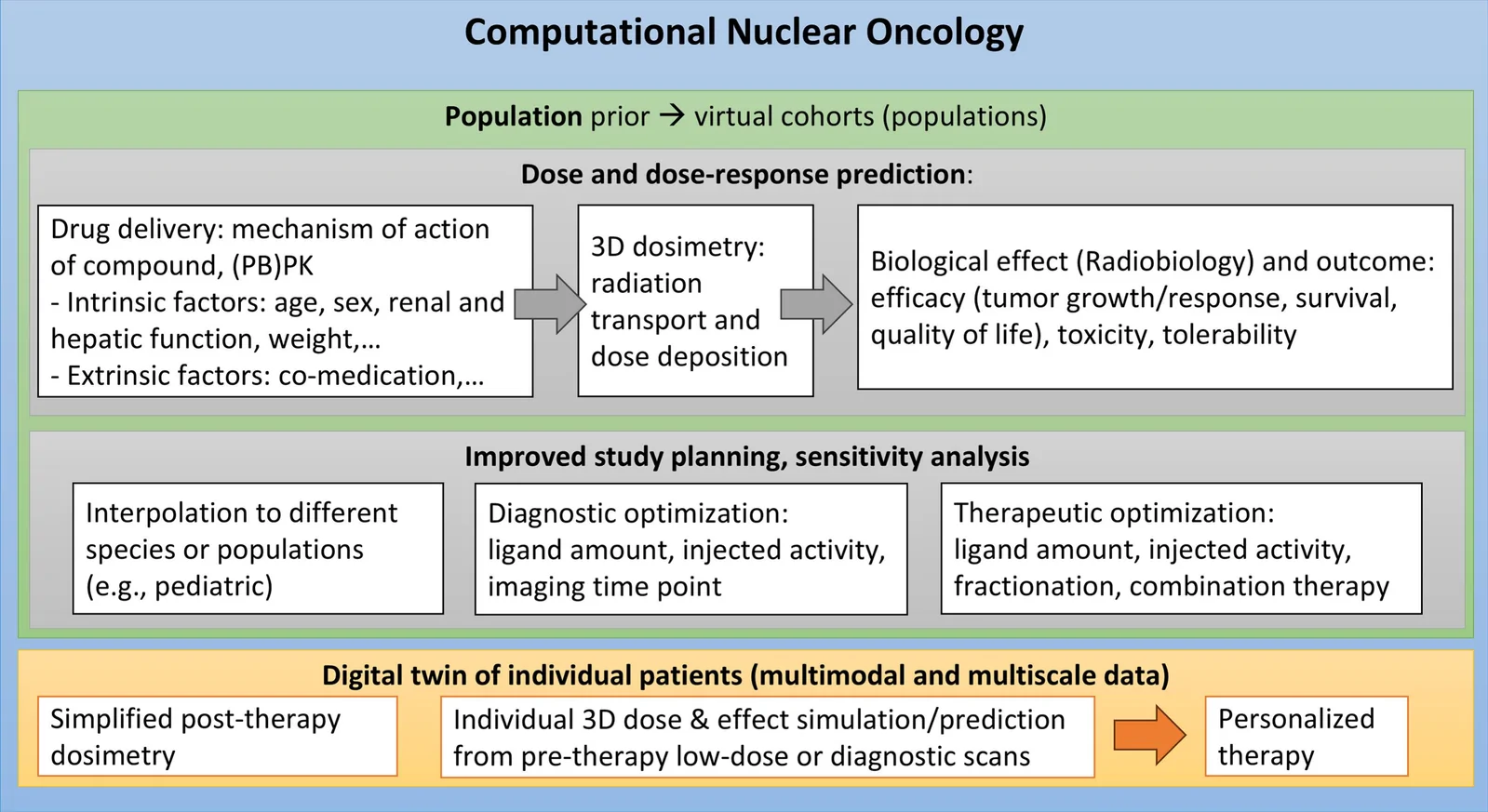
Framework of computational nuclear oncology for radiopharmaceutical therapy.

Computational nuclear oncology provides a framework for linking population-based data with individualized treatment planning (Fig. 7). Starting from population priors that generate virtual cohorts, drug delivery, pharmacokinetics, and dose deposition can be combined with radiobiological models to predict therapeutic efficacy, toxicity, and tolerability. Beyond outcome prediction, the approach supports study design and sensitivity analyses, enabling the optimization of diagnostic and therapeutic strategies, as well as the extrapolation of findings across different populations. By integrating multimodal and multiscale data into digital twins of individual patients, computational nuclear oncology paves the way toward personalized dosimetry, pre-therapy dose forecasting, and ultimately individualized radiopharmaceutical therapy [179], [180], [181], [129], [130], [182], [183], [184], [185], [186].

### Post-therapeutic dosimetry

In radiopharmaceutical therapy, post-therapeutic imaging remains the cornerstone of individualized dosimetry. Following administration of the therapeutic radiopharmaceutical, serial quantitative SPECT or PET acquisitions are performed to capture the time-dependent uptake and clearance in tumors and organs at risk. These measurements form the basis for constructing time-activity curves (TACs), to which kinetic models are fitted, ultimately yielding time-integrated activity (TIA) and subsequently the absorbed dose estimates for critical organs and lesions [187], [188]. Post-therapeutic dosimetry is then used to inform and optimize the dosing of subsequent therapy cycles.

Traditionally, post-therapeutic dosimetry involves multiple labor-intensive steps, including quantitative image reconstruction, segmentation, curve fitting, and dose calculation at either the organ or voxel level. Each of these stages introduces operator-dependent variability while consuming considerable clinical time. To address these challenges, artificial intelligence (AI) has emerged as a transformative strategy, with the potential to streamline the dosimetry pipeline, reduce workload, and improve reproducibility. AI-driven approaches are increasingly applied to dosimetry-related tasks such as automated organ and tumor segmentation, image registration, and quantitative imaging [189]. Kinetics need to be accurately described for dosimetry. While simplified mono- or bi-exponential functions are commonly used, these approaches can be refined by applying more advanced methods such as population-based pharmacokinetic analysis with non-linear mixed-effects (NLMEM) modeling or by integrating AI with PBPK models. Such strategies allow a more robust and biologically informed characterization of radiopharmaceutical kinetics, ultimately improving the reliability of dosimetry.

#### Simplified dosimetry

One of the major challenges in dosimetry is the need for multiple post-therapy imaging sessions, which poses significant logistical demands and can be burdensome for patients. Consequently, considerable efforts have focused on reducing the number of imaging time points via simplified dosimetry methods. Among these strategies, single-time-point (STP) dosimetry has gained increasing attention as a pragmatic alternative. To improve the accuracy and reproducibility of STP methods, and to create a reference model, population-based model selection (PBMS) and NLMEM have been proposed [190], [191]. These approaches leverage information across patients, thereby increasing the effective data-to-parameter ratio, improving model identifiability, and reducing the risk of overfitting compared with individual curve fitting. At the population level, statistical model selection further enhances reproducibility by identifying robust reference functions, in contrast to heuristic model-choice strategies.

Recent studies demonstrated that STP dosimetry informed by PBMS NLMEM and anchored by population-based reference models can yield reliable organ dose estimates with substantially reduced imaging burden [190], [191]. These methods outperformed commonly used STP strategies, such as those introduced by Hänscheid and Madsen [192], [193]. Importantly, the application of population pharmacokinetic modeling with NLMEM is consistent with regulatory guidance from the U.S. Food and Drug Administration (FDA) [194]and European Medicines Agency (EMA) [195], which endorse nonlinear mixed-effects approaches as powerful tools for analyzing sparse data, a scenario particularly common in nuclear medicine dosimetry.

An AI approach based on machine learning for calculating the TIA using STP data has also been proposed: Gomes et al., reported a machine learning-based STP method that achieved a maximum mean absolute percentage error (MAPE) of 27% from all investigated time points and suggested that imaging could be performed at any time point for STP dosimetry [196] . While this represents an innovative step, caution is warranted in interpreting these findings. First, a population-level MAPE of 27% implies that individual patients may still experience substantial errors, which is clinically significant in therapy planning. Second, the use of 27% MAPE as a benchmark is debatable, as no consensus currently exists on acceptable accuracy thresholds in STP dosimetry. Other work adopted a stricter criterion of 10% RMSE [190], [191]. These differences underscore the urgent need for consensus on standardized accuracy metrics for STP dosimetry if such methods are to be widely adopted in clinical practice. Ultimately, the requirements for acceptable STP accuracy will be determined by the shape of the absorbed dose–effect relationship.

Beyond statistical frameworks such as NLMEM for optimizing dosimetry, physics- and biology-informed modeling approaches are being developed to directly learn the radiopharmaceutical pharmacokinetics. Here, PBPK models provide a mechanistic bridge, accounting for intrinsic factors such as age, sex, body weight, co-meditation, and renal or hepatic function [197]. This “middle-out” strategy combines mechanistic insight with data-driven learning, making it particularly valuable in radiotheranostics, where datasets are often sparse and heterogeneous [198].

#### Absorbed dose distribution

Finally, translating 3D activity maps into 3D absorbed dose distributions remains computationally demanding. Although Monte Carlo simulations are regarded as the gold standard, their clinical use is constrained by runtime. Surrogate approaches, such as voxel S-value kernels, offer faster computation but often struggle with heterogeneous tissue geometries. Recently, deep learning-based surrogates have shown promise in overcoming these limitations. Lee et al., and Akhavanallaf et al., demonstrated that deep learning can generate voxel-level dose maps orders of magnitude faster than Monte Carlo simulations, while also surpassing kernel-based methods in accuracy [199], [200]. These advances represent a paradigm shift toward patient-specific dosimetry workflows that are both accurate and clinically feasible. Predictive dosimetry and pre-therapy dose forecasting

While post-therapy dosimetry provides valuable retrospective evaluation, the ability to predict absorbed dose prior to the first therapy cycle, or pre-therapy dosimetry, is highly desirable, as it enables patient-specific activity prescription before treatment even begins. Such prospective planning stands in contrast to the conventional paradigm of post-therapeutic dosimetry, where the first cycle is typically administered with a fixed activity [201], [202], [179], [180]. This consideration is particularly critical for agents with narrow therapeutic indices, such as [^177^Lu]Lu-PSMA-617, where optimization of the administered activity and mass could directly impact both safety and efficacy.

A common strategy is to perform pre-therapeutic imaging using diagnostic analogs, for example, [^68^Ga]Ga-PSMA-11 prior to [^177^Lu]Lu-PSMA-617 therapy, taking advantage of the superior quantitative accuracy of PET, which offers lower uncertainty than SPECT or planar scintigraphy [203], [186]. However, direct extrapolation of diagnostic tracer kinetics to therapy is inherently challenging. Differences in half-life, administered activity, as well as pharmacologic conditions limit direct extrapolation from diagnostic to therapeutic tracers. In particular, PET radionuclides such as ^68^Ga or ^18^F have short half-lives and are injected at lower masses, which restricts the imaging window and often leads to pharmacokinetics that deviate substantially from those of therapeutic isotopes such as Lutetium-177 or Actinium-225.

**Fig. 8:**
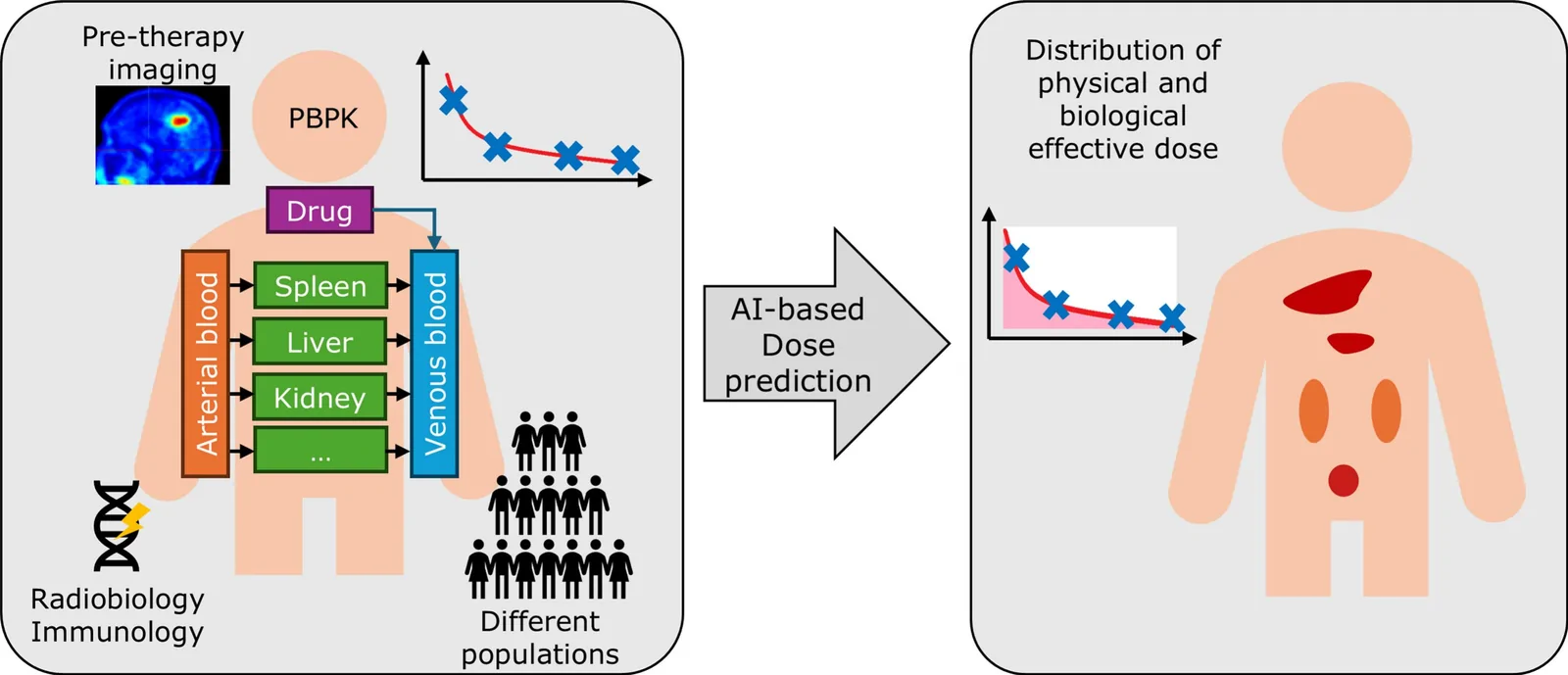
Predictive dosimetry using AI and PBPK models linking pre-therapy PET imaging with therapeutic absorbed dose predictions, enabling patient-specific activity prescription before radiopharmaceutical therapy initiation.

To address these limitations, PBPK models and AI have been proposed as tools to bridge diagnostic-to-therapeutic translation (Fig. 8). PBPK models offer mechanistic corrections by incorporating physiological and physicochemical determinants of tracer kinetics, whereas AI approaches can capture complex relationships between pre-therapeutic PET kinetics and therapeutic absorbed doses. Proof-of-concept in silico studies have already demonstrated the feasibility of PET-based treatment planning using PBPK modeling, such as predicting absorbed doses for [^90^Y]Y-DOTATATE therapy from [^68^Ga]Ga-DOTATATE PET kinetics [203].

Despite this promise, the accuracy of PET-based predictions remains limited when relying solely on measured diagnostic kinetics. For instance, attempts to predict [^177^Lu]Lu-HA-DOTATATE kinetics from [^68^Ga]Ga-HA-DOTATATE PET imaging reported mean absolute percentage errors (MAPEs) of up to 39% [204]. Multicenter studies correlating baseline PET renal uptake with absorbed kidney doses from [^177^Lu]Lu-DOTATATE therapy yielded MAPEs of up to 28% [205]. Even greater discrepancies have been observed for [^177^Lu]Lu-PSMA-617, where extrapolation from [^68^Ga]Ga-PSMA-11 PET/CT biokinetics resulted in errors of ∼40% for kidneys and up to 170% for tumors [202]. These findings underscore the risks of direct extrapolation and highlight the need for approaches that integrate mechanistic and statistical rigor.

Hybrid frameworks combining pharmacokinetic models with AI are therefore gaining momentum. For example, Kassar et al. [150] embedded PBPK constraints within a deep learning architecture, thereby regularizing voxel-wise dose predictions with mechanistic priors [150]. Such strategies not only enhance robustness but also address the growing demand for explainable AI in clinical practice. Beyond correction, AI methods can reveal unknown relationships between diagnostic PET parameters and therapeutic absorbed doses, enabling more reliable extrapolation across patient populations and imaging settings. Taken together, these developments point toward the necessity of standardized frameworks that integrate diagnostic PET with PBPK- and AI-based predictive models. Establishing such methodologies will be essential for advancing PET-based pre-therapy dosimetry and ultimately enabling personalized activity prescription in clinical radiopharmaceutical therapy.

### Dose-response modeling and biological effect prediction

Incorporating biological effects into dosimetry represents a critical next step toward fully personalized radiopharmaceutical therapy. Optimizing treatment requires not only estimating absorbed doses but also predicting biological outcomes such as tumor control probability, toxicity risk, and patient-reported quality of life. Traditional dose-response analyses in external beam radiotherapy rely on linear-quadratic models with population-averaged parameters. In RPT, however, the continuous temporal distribution of activity and the stochastic nature of radionuclide decay demand radiobiology models tailored to these unique characteristics. In addition, factors such as non-uniform, small-scale activity distribution at the cellular and subcellular level, cross-dose contributions from surrounding structures, heterogeneous radiosensitivity, prior therapies, organ function, and treatment timing further complicate the dose-effect relationship and must be explicitly considered in model development. This limitation constrains purely correlative models trained on sparse retrospective data and motivates computational nuclear oncology approaches that integrate dosimetry, pharmacokinetics, radiobiology, and multimodal covariates in order to move toward mechanistically informed, patient-specific inference.

Recent work has started to bridge dosimetry and outcome modeling by combining absorbed-dose metrics with additional covariates in AI-based prediction frameworks. For example, Wei et al., used lesion-level absorbed-dose metrics together with radiomic features to predict lesion response and progression after radioembolization [206], whereas Mansouri et al., incorporated dosimetry-derived dose-volume histograms, radiomics/dosiomics, and baseline biomarkers to predict survival and short-term tumor response after personalized radioembolization [207]. These studies support the feasibility of multimodal dose-response modeling, but they also highlight that the field is still at an early stage, with small datasets and predominantly retrospective, correlative analyses rather than prospective, mechanistically informed prediction.

The computational nuclear oncology concept offers a powerful means of addressing this complexity. Dose kernels can be coupled with voxel-level radiobiological models that incorporate DNA-damage repair kinetics, enabling end-to-end inference of both physical dose and expected biological effect within a single computational step [208], [209], [210], [211]. Models that correlate delivered dose with biological effect, such as tumor volume regression, overall survival, and quality of life measures, may enable outcome-driven activity prescriptions that improve tumor control probability [212], [213], [214], [215].

Kletting et al., demonstrated that PET/CT-derived pharmacokinetic features could predict tumor response, illustrating the potential of imaging-driven predictors in guiding therapy [216]. Together, these advances signal a shift from administered activity-based planning to biology- and outcome-driven optimization, an essential step in the evolution of precision RPT.

Realizing this vision in routine practice will require rigorous prospective verification, validation, and uncertainty analysis, harmonization of biological endpoints, and engagement with regulatory bodies. Such efforts will be crucial to ensure that biologically informed dosimetry not only improves predictive accuracy but also translates into safer and more effective treatment strategies.

### Digital twins for personalized radiopharmaceutical therapy

In contrast to external beam radiotherapy, where individualized dose planning is routine, RPT still commonly follows a fixed-activity “one-size-fits-all” paradigm. This approach risks under-treatment in some patients and excessive toxicity in others, ultimately limiting both therapeutic efficacy and quality of life. Digital twins (DTs) have emerged as a potential solution, offering computational replicas of individual patients that can simulate therapy scenarios in silico. By capturing the patient’s physiological, pharmacological, and imaging-derived characteristics, DTs enable prediction of absorbed dose and treatment response under varying conditions. This allows virtual testing of different activity levels, injection schemes, or even combination regimens before they are administered clinically [129], [130].

Beyond individual treatment optimization, DTs can be used to create virtual cohorts representing diverse patient populations. These cohorts provide a framework for testing novel radiopharmaceuticals, designing clinical trials, and evaluating inter-patient variability without the immediate need for extensive preclinical or animal studies. Realizing the potential of DTs requires continuous integration of multimodal patient data. This includes physiological parameters, molecular imaging biomarkers, genomic or pharmacogenomic profiles, and radiobiological descriptors such as DNA repair capacity or microenvironmental features. Incorporating these datasets into PBPK and NLMEM frameworks, and augmenting them with AI-driven inference, provides the mechanistic and statistical foundations for DT construction. Importantly, DTs are not static models; they must be dynamically updated as new clinical and imaging data become available, allowing them to evolve in parallel with the patient’s treatment course [217].

The clinical deployment of DTs will depend on robust verification, validation, and uncertainty quantification [184], [214], [215], as well as harmonization of data standards and interoperability across institutions. DTs could shift RPT from population-based dosing to adaptive, feedback-informed strategies, where therapy is adjusted in real time to maximize efficacy and minimize toxicity. While challenges remain in data availability, computational complexity, and regulatory acceptance, DTs represent a logical next step in the integration of AI, PBPK, and NLMEM into clinical radiopharmaceutical therapy, moving the field closer to truly individualized precision treatment.

## Preclinical applications

Unlike clinical imaging, preclinical studies involve much smaller structures and faster physiology, posing challenges for preserving quantitative accuracy in pharmacokinetics, dosimetry, and therapy response endpoints. Recent years have seen a rapid increase in AI applications across small-animal imaging pipelines.

### Reconstruction for dose- and time-efficient small-animal PET/SPECT

DL denoising and image-to-image synthesis allow substantial reduction in injected activity or scan time in micro-PET while preserving quantitative accuracy. Lowering injected activity is of a special importance in mouse PET/SPECT imaging, as commonly used preclinical radiation doses were demonstrated to produce deterministic alterations in biological pathways [218]. Muller at al. trained a 2D U-Net on high-low count [^18^F]FDG-PET image pairs, reported a superior denoising performance of the DL model compared to spatial and edge-preserving denoising filters, and successfully translated the framework to other tracers [219]. Similarly, Dutta et al., developed and evaluated a novel DL architecture, Attention-based Residual-Dilated Net (ARD-Net), to generate high-count mouse PET from low-count inputs [120], [121]. They verified with task-based assessments, that lesion detectability and quantitative metrics were maintained versus reference reconstructions. Comparable gains have been demonstrated when learning in the sinogram domain for preclinical low-dose PET, where DL models achieved better denoising performance than conventional algorithms [220].

### Whole-body organ and tumor segmentation

The lack of standardized methods for defining VOIs in preclinical PET/CT severely impacts data reproducibility [221], [222]. Automated segmentation would enable reproducible image quantification; however, the literature on preclinical segmentation is scarcer than on clinical. One of the existing solutions is AIMOS (AI-enabled Mouse Organ Segmentation), which provides near-real-time multi-organ labeling on whole-mouse CT scans, drastically reducing manual effort. Another group [113], [114], [115], [116] benchmarked a Swin UNEt TRansformer (Swin UNETR) [223] against the AIMOS model and the 3D nnU-Net [97] on native and contrast-enhanced micro-CTs, with external-site validation demonstrating robustness across scanners and protocols. In this study, Swin UNETR was shown to be superior to the other two models; however, the results should be interpreted with caution to avoid the known bias towards novel architectures [224]. These AI tools support emerging disease-specific preclinical segmentation pipelines; for instance, Sun et al., developed automatic bone segmentation from micro-CT slices using an attention U-Net to track myeloma-related bone marrow changes [225]. In nanomedicine pharmacokinetics, the NanoMASK (Nanomedicine Multimodal AI-based Segmentation for PharmacoKinetics) 3D U-Net segments multiple mouse organs and tumors on PET/CT time series and outputs dosimetry-relevant metrics automatically [226].

### Quantitative dynamic PET without arterial sampling

Obtaining an arterial input function in mice is invasive and often impractical. Thus, inferring the input function directly from dynamic PET is a highly relevant task. Kuttner et al., trained a DL model that predicts the arterial input function from dynamic [^18^F]FDG mouse PET images, enabling kinetic parameter estimation without arterial sampling [221], [222]. Such approaches reduce complexity of the preclinical pipeline while opening routine access to pharmacokinetic modelling in small-animal studies.

### Radiomics

Preclinical PET/CT radiomics enables hypothesis-driven biomarker discovery under controlled biology. For instance, in metastatic non-small cell lung cancer mouse model, PET radiomics captured the dynamics of tumor-infiltrating CD8^+^ T-cell exhaustion after ablative irradiation and supported timing decisions for combining radiotherapy with immune checkpoint inhibitors (ICI) treatment [29], [30]. Roy et al., were able to predict the response to neoadjuvant chemotherapy in triple-negative breast cancer in both preclinical and clinical cases using robust radiomics features as an input for shallow learning models [227]. Benfante et al., studied a novel [^64^Cu]-labeled chelator by PET in a mouse model at different timepoints to evaluate its biodistribution [228]. Radiomic features automatically extracted from whole-body mouse scans were compared between the timepoints, indicating bladder and liver as the organs with the highest observed variation. The authors highlight high potential of radiomics for preclinical biodistribution studies.

### Outlook

Thus, in preclinical nuclear medicine, AI enables low-dose PET/SPECT, automated small-animal segmentation, noninvasive pharmacokinetic modeling, and smarter tracer development pipelines. However, open and well-annotated preclinical datasets, as well as shared models, remain scarce, hindering method validation and reproducibility. Nonetheless, ongoing efforts are being made in this area [229], [230].

## Development of new radiopharmaceuticals

Beyond clinical application, AI is increasingly being harnessed to accelerate the discovery and optimization of novel radiopharmaceuticals, helping to prioritize radiotracer candidates before costly in vivo testing. AI models can screen large chemical libraries, optimize binding affinities, and simulate pharmacokinetics under radiochemistry-specific constraints [231], [232]. Dedicated models such as DeepDelta [233] can predict molecular property differences based on the ADMET (absorption, distribution, metabolism, excretion, and toxicity) score. Other models have been employed to determine blood-brain barrier permeability of CNS tracer candidates [234], [235]. Machine learning approaches have also been proposed for in silico central nervous system radiopharmaceuticals design prior to small-animal trials, focusing on reinforcement learning to iteratively optimize candidate molecules toward higher success rates [236]. Comparable strategies have already proven effective in accelerating vaccine development, complementing simulations with virtual compound design [237]. AI has also been proposed for identifying theranostic ligands with favorable kinetic and toxicity profiles [6]. Similarly, Xu et al., identified potential theranostic target structures by cross-referencing large genome and protein databases [238].

When integrated with PBPK simulations, these approaches enable virtual assessment of candidate biodistribution, allowing prioritization of compounds before synthesis and preclinical testing. In line with the FDA’s Roadmap to Reducing Animal Testing in Preclinical Safety Studies [239], model-informed drug development, including AI, population pharmacokinetics, and PBPK, plays a central role in reducing reliance on animal studies while improving translational efficiency. The convergence of AI, PBPK, and NLMEM thus establishes a powerful foundation for in silico radiopharmaceutical discovery. This integrated framework not only enables rapid screening of molecular candidates for finding the optimal affinity and biodistribution but also ensures regulatory alignment with best practices in pharmacometrics. Such synergy has the potential to shorten development cycles, reduce costs, and overcome translational hurdles, ultimately accelerating the availability of safer and more effective theranostic agents.

## Regulatory aspects

Although the number of research publications on AI in medical imaging and therapy in nuclear medicine is almost overwhelming, the number of approved applications (either by the FDA or by its European entities) is relatively moderate. The list of AI and ML enabled medical devices [240] – which actually includes software since almost a decade – has currently 1249 entries, where SPECT and PET acquisition-related entries contribute less than 2% and analysis software an even lower percentage (<0.5%). Not unexpected, radiology related devices contribute 76%. For Europe, healthairegister.com, lists currently 265 products where nuclear medicine is targeted by less than 5% of all listed (software) devices. The reason for this gap between research and products is the complexity of the approval process governed by the European AI act (artificialintelligenceact.eu) and the Medical Device Regulation (MDR), which includes Software as Medical Device (SaMD). This regulatory complexity, together with the need of validation and prove of scientific evidence, is fortunately increasingly addressed – although radiological applications dominate [241], [242], [243], [244]. That a strict regulatory process is needed stands without question given the relevance of the topic, and its importance is further enhanced by a very rapidly evolving technological landscape [243], [244].

## Future perspectives

Although radiomics and conventional machine learning (ML) approaches have been frequently applied in nuclear medicine, to date, they have not been widely adopted in clinical routine, mainly due to their lack of generalizability. Feature harmonization strategies have therefore been proposed [245], [246], [247]. The application of convolutional neural networks in nuclear medicine has shown exponential growth with broad utilization for object detection and segmentation, as well as for diagnosis and prognostication [2]. Yet, nuclear medicine operates with relatively small, heterogeneous, and imbalanced datasets, inherently challenging the successful utilization of state-of-the-art AI models [92]. Deep learning, using e.g., CNNs, requires large, multicentric data and shared data processing platforms to operate with, which nuclear medicine struggles to deliver. Future solutions need to rethink the AI strategy of nuclear medicine at its core in order to be able to apply AI-based methods effectively.

### Foundation models and agentic AI

Furthermore, so-called foundation models have been proposed to combine multimodal data for modeling complex dynamic processes and ultimately, to predict clinical endpoints from multimodal data. Currently, there is an ongoing debate about the actual usefulness and/or computational efficiency of these approaches [118].Oh et al., applied a foundation model to leverage the utility of a multitask general-purpose solution for PET/CT data by using data from the AutoPET challenge [248].

Large language models are foundation models for text generation. They can draft structured reports, generate imaging protocols, and assist in troubleshooting quality‑control deviations, resulting in time savings but raising concerns about hallucinations. When certain conditions apply (e.g., medical-specific large language models (LLM) or properly set-up LLM with medical Retrieval-Augmented Generation approaches), LLMs can be useful tools for data retrieval and for generating structured information about reports for further use.

There are foundation models that are not language but image-based, e.g., utilizing vision transformer architecture (ViT) [249]. The utility of these approaches to date has not been demonstrated widely as ViTs suffer from the same limitation as DL or CNNs: while ViT within foundation models does not require labeled images, the lack of large datasets for training limits its application in the domain of nuclear medicine. Bradshaw et al., suggested a combination of large language models with medical imaging [250]. A typical task would be, for example, to give the model the task: write a radiological report on this image, or to tell the model where a lesion is, which the model would then segment.

Another recent trend goes even beyond LLMs by introducing so-called AI agents. These agents should enable seamless communication, reasoning, decision-making, and task execution using available tools, with foundation models serving as their cognitive core [251], [252].

This is an active research area which - partly due to the above challenges - requires thorough investigation of LLM, ViT, or hybrid, multi-modal foundational models, preferably in prospective clinical trial settings.

### Biomorphic AI

Another approach is “Biomorphic AI”, which is based more on biological organisms and is therefore potentially more robust and flexible. To date, the most typical biomorphic AI approaches are based on spiking neural networks (SNN) that mostly build on the so-called Hebbian rule (neurons that fire together, wire stronger together)[253]. However, recent proceedings in fundamental brain research debunked the Hebbian rule in biological organisms [254], [255]. In addition, no superior SNN has been demonstrated over conventional artificial NNs (ANNs)[253]. This implies that the core logic of SNNs may need to be rethought to make them widely powerful AI tools. In contrast to this, any trained ANN can be converted into SNNs, and such systems are ideal to host them on-chip [256]. These solutions recently ignited a new wave of analogue AI chip revolution, as their efficient, on-premise, and fast inference capabilities clearly demonstrate an advantage over ANNs that require graphics processing units (GPUs) for inference. The necessity of having very fast inference of deployed AI in nuclear medicine is not a generic requirement, but rather a specific one. While prediction does not need to be real-time, an AI system deployed on-chip as part of an image acquisition system where AI-based resolution recovery or low-dose to high-dose AI tasks are required, gives a clear advantage and credibility to rely on such solutions.

### Quantum AI

Quantum computing has fundamental properties (superposition and entanglement) that allow us to model and manipulate complex tasks with a low number of quantum bits (qubits) with an unprecedented level of speed [257]. Specifically, one can encode N classical numbers (e.g., radiomic features or image voxels) into log2N number of qubits. On that low qubit count, one can build a quantum AI which – in the realms of nuclear medicine use-cases – requires only a couple hundreds of unknown parameters to train [258]. Initial trials suggest that this encoding step, along with the required low trainable parameter count, can significantly increase quantum AI predictive performance and generalization abilities [259]. Various findings have been presented that demonstrate a quantum advantage is possible in simulator environments, which points toward the economically feasible utilization of quantum logic in the field of quantum AI, particularly when relying on nuclear medicine data.

## Discussion

The rapid expansion of artificial intelligence in nuclear medicine highlights both opportunities and challenges. Technically, AI has already surpassed conventional methods in several domains, including coincidence time-resolution estimation, denoising, automated lesion detection, and tracer kinetic modelling. In translational research, AI is accelerating radiopharmaceutical discovery, enabling predictive dosimetry, and paving the way for digital twins that simulate therapy scenarios before treatment. Together, these developments signal a paradigm shift toward data-driven, individualized nuclear medicine.

However, widespread clinical adoption remains limited. Four overarching challenges can be identified. First, generalizability: most AI models are trained on single-center or homogeneous datasets, making their performance across scanners, institutions, and diverse patient populations uncertain. While nested cross‑validation on internal data mitigates optimistic bias, external testing on unseen scanners remains indispensable. Federated learning and harmonization frameworks hold promise but require broader implementation, e.g., by multicentric approaches [4], [5]. Second, reference data: robust, standardized datasets remain scarce, and without them, reproducibility and benchmarking are difficult. Furthermore, manual data labeling for supervised learning may be labor-intensive and introduce biases and errors [27]. Although digital phantoms, Monte Carlo simulations, and phantom measurements offer valuable surrogates, community-level efforts are needed to establish shared repositories within the community. Third, trust and transparency: explainability, continuous monitoring, and precise uncertainty estimates are prerequisites for regulatory approval and clinical confidence [260]. Fourth, digitization: digitally available data is a prerequisite for the use of AI. However, much of the data in German hospitals is still collected on paper or as a mixture of paper and electronic formats [261].

Effective communication across the community is essential to overcoming these barriers. Progress depends not only on collaboration between AI developers and physicians but also on systematically incorporating patient perspectives. Patients are the ultimate stakeholders and should be involved early in evaluating whether new tools meet clinical needs. Joint symposia that bring together developers, clinicians, regulators, and patient representatives can ensure that innovations are aligned with practical requirements and patient priorities rather than driven solely by technical feasibility.

Education and training also play a central role. Nuclear medicine specialists must develop literacy in AI methods, while AI researchers must better understand clinical workflows, data constraints, privacy issues including data protection, and regulatory realities. Only by fostering a shared language across disciplines can the field translate promising prototypes into trusted clinical products.

Beyond technical performance and communication, reimbursement and implementation economics will strongly influence whether AI tools enter routine nuclear medicine practice. Even when an algorithm improves efficiency by automating time-consuming steps, adoption will remain limited unless data curation, AI implementation, validation, and maintenance, and user training costs are balanced by reimbursement pathways or demonstrable cost-effectiveness. In practice, the value proposition of AI must therefore be assessed not only by accuracy gains but also by its impact on workflow time, staffing requirements, report quality, and downstream clinical decision-making.

Finally, the regulatory landscape demands careful navigation. The European AI Act, the Medical Device Regulation (MDR), and FDA or EMA guidance all emphasize rigorous external validation, post-market surveillance, and continuous performance auditing [262]. In this context, model cards are an important tool for compliance with the EU AI Act and serve as a standardized form of documentation to ensure that AI models are transparent, secure, and trustworthy. They provide information such as model details, intended use, training data, evaluation data, results, ethical considerations, risk metrics, and offer regulatory authorities and users key insights into the purpose, capabilities, and limitations of the models. Harmonization of standards, such as RELAINCE [91], the joint radiomics guideline of EANM and SNMMI [92], [263] (IBSI), CLEAR [93], and CLAIM [94], will be crucial for ensuring that AI tools are reproducible, transparent, and clinically meaningful [6].

## Conclusion

Artificial intelligence techniques have already surpassed traditional methods in several applications in nuclear medicine. Yet, widespread clinical adoption will require consensus on reference datasets, rigorous external validation, benchmarking against standards, implementation of quality control measures, federated infrastructure, clear explanations of algorithmic decisions, defined responsibility structures, protection against bias, and safeguards for data privacy. For clinical decision support, human oversight remains essential, particularly when models are continuously updated or trained on heterogeneous multicenter data [6]. The latter is yet another challenge as deskilling may also play a role in AI-integrated clinical workflows. Taken together, the integration of AI into nuclear medicine is not simply a technological challenge but a societal one. Its success will depend on sustained collaboration between physicists, computer scientists, clinicians, regulators, and patients, with the shared goal of developing trustworthy tools that truly improve patient care.

## CRediT authorship contribution statement

**Lena Kaiser:** Writing – review & editing, Writing – original draft, Conceptualization. **Thomas Wendler:** Writing – review & editing, Writing – original draft. **Deni Hardiansyah:** Writing – review & editing, Writing – original draft. **Dirk Hellwig:** Writing – review & editing, Writing – original draft. **Philipp Lohmann:** Writing – review & editing, Writing – original draft. **Stephan Nekolla:** Writing – review & editing, Writing – original draft. **Laszlo Papp:** Writing – review & editing, Writing – original draft. **Philipp Ritt:** Writing – review & editing, Writing – original draft. **Lalith Kumar Shiyam Sundar:** Writing – review & editing, Writing – original draft. **Johannes Tran-Gia:** Writing – review & editing, Writing – original draft. **Artem Zatcepin:** Writing – review & editing, Writing – original draft. **Isabelle Miederer:** Writing – review & editing, Writing – original draft, Conceptualization.

## Declaration of competing interest

The authors declare the following financial interests/personal relationships which may be considered as potential competing interests: LK and PR are employees at ITM Oncologics GmbH. LKSS is co-founder of Zenta GmbH, Vienna, Austria. PL is a member of the advisory board of Servier Pharmaceuticals and received speaker honoraria from Blue Earth Diagnostics and is a consultant for Telix Pharmaceuticals. This publication was written independently of the mentioned companies.
